# Behavioral and neural correlates of self-referential processing deficits in bipolar disorder

**DOI:** 10.1038/srep24075

**Published:** 2016-04-07

**Authors:** Yanli Zhao, Wenbo Luo, Jingxu Chen, Dandan Zhang, Ligang Zhang, Chunling Xiao, Fengmei Fan, Xiaolin Zhu, Hongzhen Fan, Shuping Tan

**Affiliations:** 1 Center for Psychiatric Research, Beijing Huilongguan Hospital, Beijing 100096, China; 2 School of Psychology, Liaoning Normal University, Dalian 116029, China; 3 Institute of Affective and Social Neuroscience, Shenzhen University, Shenzhen 518060, China

## Abstract

Self-referential processing is a core component of social cognition. However, few studies have focused on whether self-referential processing deficits present in bipolar disorder. The current study combined a high-time-resolution event-related potential (ERP) technique with the self-referential memory (SRM) task to evaluate self-referential processing in 23 bipolar patients and 27 healthy controls. All subjects showed a reliable SRM effect, but the bipolar group had poorer recognition scores for the self- and other-referential conditions. The bipolar group presented with smaller voltages in both the self- and other-referential conditions for the N1 (150–220 ms) and the P2 components (130–320 ms) but larger voltages in the positive slow wave (600–1600 ms) component. Larger P3 amplitudes were elicited in the self-referential condition compared with the other-referential condition in controls but not in bipolar patients. Additionally, non-psychotic bipolar patients had a comparative normal SRM effect which was abolished in psychotic bipolar patients; non-psychotic bipolar patients had larger amplitudes of the positive slow wave than the normal controls, whereas it was not differed between psychotic bipolar patients and the healthy subjects. The present study suggests that self- and other-referential processing is impaired in bipolar patients and the deficits may be more pronounced in psychotic bipolar patients.

Self-referential processing (or self-reflection) refers to conscious decision making regarding matters related to the self^1^. Self-referential processing is a core component of self and social cognition^2^,^3^ and is crucial for social adaptation^4^.

“Self-disorder”has been traditionally considered a core feature of schizophrenia^5^; however, less focus has been placed on whether such deficits are also present in other mental disorders. One exception was a study conducted by Moe and Docherty^6^ in which a disrupted sense of self was assessed from spontaneous narratives provided by patients with schizophrenia and psychotic bipolar disorder, as well as normal control participants. Results revealed that certain aspects of agency and relatedness to others were deficient in schizophrenia compared to that in psychotic bipolar disorder; the groups were comparable on other aspects of the self, including differentiation, substantiality, articulation and quality of relatedness, striving/ambition, and self-criticism. It is well known that one’s sense of self is a complex construct and is often conceptualized differently. Thus, it may be difficult to explain relationships between a sense of self in Moe and Docherty’s study and aspects of self-referential processing assessed in the present study. Nevertheless, Moe and Docherty observed that certain aspects of one’s sense of self were disrupted specifically in schizophrenia while others were related to psychosis.

The goal of the present study was to explore self-referential processing deficits in bipolar disorder from a cognitive neuroscience perspective. Specifically, we aimed to extend prior research by exploring possible self-referential processing decrements in bipolar disorder using a self-referential memory (SRM) task and an event-related potential (ERP) technique. Literature assessing self-referential processing deficits has focused on schizophrenia, and most of these studies have employed functional magnetic resonance imaging (fMRI) techniques. Results have revealed neuronal substrates underlying self-referential abnormalities in cortical midline structures, including the medial prefrontal cortex and posterior cingulate^7^,^8^,^9^.

The SRM task is a common self-referential behavioral paradigm. The task consists of two phases^2^,^10^,^11^: encoding and recognition. The encoding stage includes three conditions: self-referential processing, other-referential condition, and a physical condition (e.g. structural processing condition). Participants are asked to judge whether a set of trait adjectives would describe themselves, other people, or to make a syntactical structure judgment regarding the adjectives, respectively. Several minutes later, an unexpected recognition phase is presented. Subjects need to judge whether the words were presented during the encoding phase. Results of assessment of healthy adult samples show that recognition scores for the self-referential condition are better than in other-referential condition, which is referred to as an SRM effect^12^. Compared with fMRI, ERP techniques are excellent for evaluating the time course of cognitive processing given ERP’s high temporal resolution^11^. ERP combined with an SRM task allows researchers to evaluate self-referential processing in bipolar disorder from both an electrophysiological and behavioral perspective.

Prior research has investigated ERP correlates of self-referential processing in healthy individuals, observing noticeable markers of processing bias toward self-relevant information, including the P300 and late positive components. For example, P300 amplitudes are larger in response to a person’s own name relative to other names^12^, and one’s own handwriting elicits more positive components than other handwriting conditions during later time windows, between 200 and 500 ms (N2 and P3 components) and 1,000–2,000 ms (LPC)^13^. Our earlier work on self-referential processing in patients with schizophrenia^11^ mainly observed that an SRM effect was absent in schizophrenia, and patients with schizophrenia demonstrated smaller positive slow wave (800–1,200 ms) amplitudes over the prefrontal cortex during self-referential conditions. Overall, the aforementioned studies discussed provide a good basis for exploring the temporal features of self-referential processing among patients with bipolar disorder.

More than half of patients with bipolar disorder experience psychotic symptoms in their lifetime^14^, and those with a psychotic history tend to have greater cognitive dysfunction^15^ and poorer prognoses^16^. Therefore, the current study also explored whether subgroups of patients with bipolar disorder would perform differently on a self-referential processing task. Based on clinical observations, patients with bipolar disorder have low and/or exaggerated self-evaluations; thus, the present study anticipated that patients with bipolar disorder would demonstrate impaired self-referential processing. Furthermore, psychotic bipolar disorder, similar to schizophrenia, is a major psychotic disorder. Therefore, it was hypothesized that self-referential processing deficits would likely be more significant in patients with psychotic features compared to those without. Relatedly, self-referential processing deficits may only be present in patients with psychotic features.

## Results

### Behavioral results

#### Encoding phase

Subjects’RTs exhibited a significant main effect of task condition [*F* (2, 96) = 103.46, p < 0.001, *η*^2^ = 0.679]. For all subjects, RTs for the font-judgment condition (mean ± standard error = 668 ± 22.8 ms) were shorter than for the other two conditions (self-referential = 1,053 ± 34.2 ms; other-referential = 1,025 ± 38.4 ms) (p < 0.001). Moreover, RTs showed a significant main effect of group [*F* (1, 48) = 26.75, p < 0.001, *η*^2^ = 0.358]. Patients responded more slowly than controls throughout the experiment (1,058 ± 40.4 vs. 773 ± 37.3 ms).

#### Recognition phase

**Figure 1:**
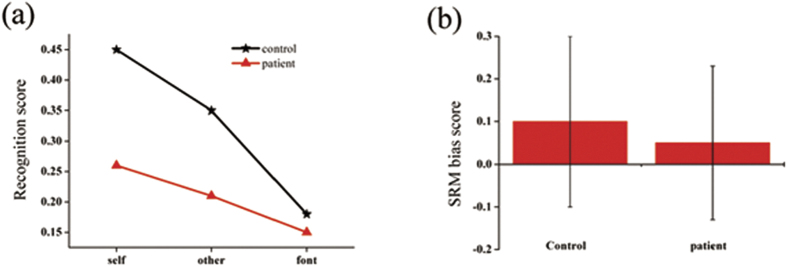
Behavioral results. (**a**) Recognition scores of bipolar patients and healthy controlsin the three experimental conditions. (**b**) Self-referential memory bias scores ofpatients and controls. Error bars correspond to two standard deviations of the mean.

The two groups demonstrated significant differences in recognition scores (task condition-by-group: [*F* (2, 96) = 11.53, p < 0.001, *η*^2^ = 0.093](Fig. 1(a)). Specifically, compared with controls, patients with bipolar disorder had lower recognition scores in both the self-referential (0.45  ± 0.03 vs. 0.26 ± 0.03, p < 0.001) and other-referential conditions (0.35 ± 0.03 vs. 0.21 ± 0.03, p < 0.001). Conversely, there were no significant differences between the two groups in the font judgment condition (p = 0.197). Moreover, both groups had higher recognition scores in the self-referential condition than in the other-referential condition (p < 0.001, p = 0.031, respectively, for controls and patients), reflecting a reliable SRM effect. However, the control group (0.10 ± 0.02) had higher SRM bias scores than the patient group [0.05 ± 0.02, *t* (48) = −1.83, p = 0.074, nearly significant] (Fig. 1(b)).

### ERP results

#### N1

**Figure 2:**
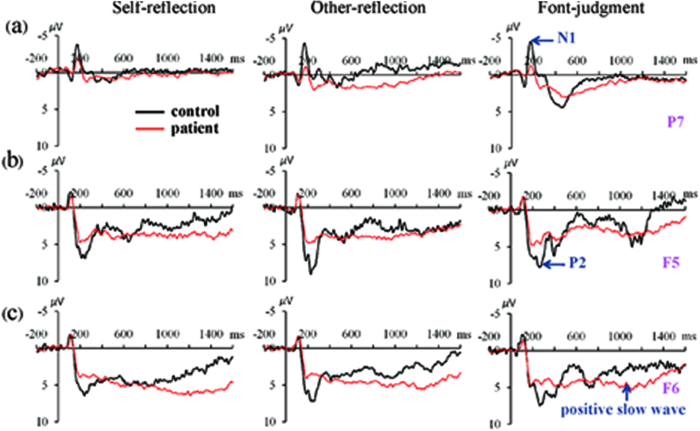
Comparisons of grand averaged waveforms between patients with bipolar disorder and healthy controls. (**a**) TheN1component at electrode site P7. (**b**) The P2 component at electrode site F5. (**c**) The positive slow waveform at electrode site F6.

**Figure 3:**
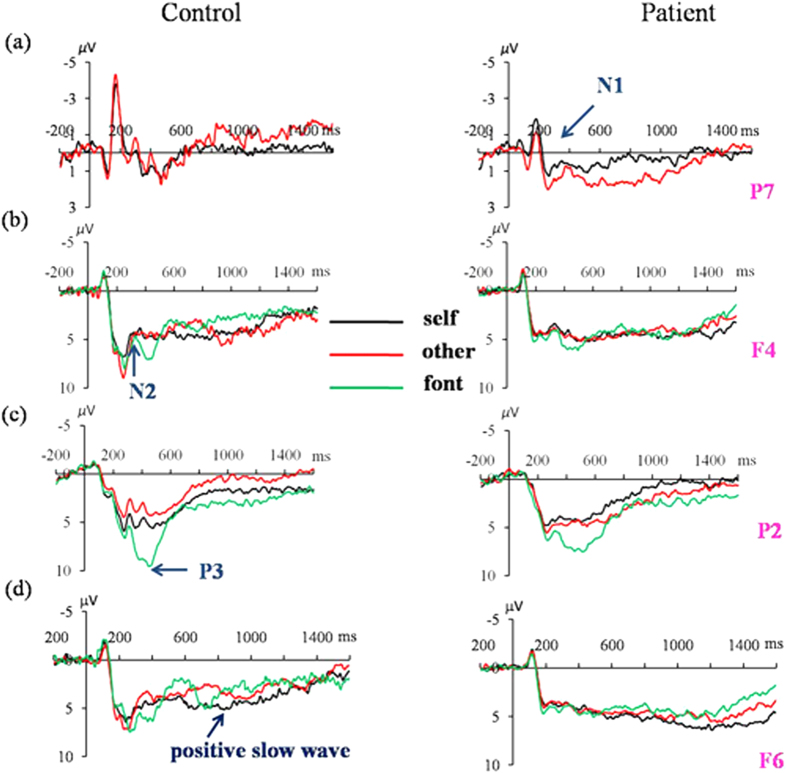
Comparisons of grand averaged waveforms between the self/other-referential and font-judgment conditions within groups. (**a**) The N1 component at electrode site P7. (**b**) The N2 component at electrode site F4. (**c**) The P3 component at electrode site P2. (**d**) The positive slow wave component at electrode site F6.

The two groups demonstrated significant differences in N1 amplitudes [electrode site-by-group-by-task condition: *F* (6, 288) = 2.54, p = 0.045, *η*^2^ = 0.048]. Specifically, the control group had a more negative deflection than the patient group, mainly at the P7 site (−4.54 ± 0.48 *μ*V vs. −2.92 ± 0.52 *μ*V, p = 0.027; −4.53 ± 0.47 *μ*V vs. −1.94 ± 0.51 *μ*V, p < 0.001; −4.90 ± 0.51 *μ*V vs. −2.32 ± 0.55 *μ*V, p = 0.001, respectively for the self-referential, other-referential, and font-judgment conditions) (Fig. 2(a)). Furthermore, for the patients, self-referencing elicited a more negative N1 component than other-referencing (p = 0.004, at electrode site P7) (Fig. 3(a)).

#### P2

P2 amplitudes revealed a significant main effect of group [*F* (1, 48) = 11.46, p = 0.001, *η*^2^ = 0.193]; P2 amplitudes were smaller in bipolar patients (6.94 ± 0.60 *μ*V) than in controls (9.70 ± 0.55 *μ*V) (Fig. 2(b)).

#### N2

N2 amplitudes showed a significant main effect of task condition [*F* (2, 96) = 4.04, p = 0.024, *η*^2^ = 0.075]. A post hoc analysis demonstrated that for all subjects, the font-judgment condition (2.61 ± 0.55 *μ*V) elicited a less negative N2 component than the other-referential condition (1.88 ± 0.52 *μ*V, p = 0.059), whereas there was no significant N2 amplitude difference between the other- and self-referential conditions (1.86 ± 0.56 *μ*V, p = 1.00) or between the font-judgment and self-referential conditions (p = 0.116) (Fig. 3(b)).

#### P3

The two groups demonstrated significant differences in P3 amplitudes (electrode site-by-group-by-task condition: [*F* (10, 480) = 2.24, p = 0.046, *η*^2^ = 0.020] (Fig. 3(c)). Specifically, font judgments elicited a more positive P3 component than both the self- (p < 0.001 at electrodes P2; p = 0.001 at CP4) and other-referential conditions in patients (p = 0.001, at electrode P2; p = 0.004, at CP4), whereas there was no significant P3 amplitude difference between the self- and other-referential condition (p = 0.763, at P2; p = 1.000, at CP4). However, the control group showed a different pattern: P3 amplitudes elicited by font judgments were larger than those elicited by self-referencing (p < 0.001, at electrodes P2 and CP4), while self-referencing elicited a more positive P3 than the other-referential condition (p = 0.042, at P2, p = 0.043, at CP4).

#### Positive slow waves

The two groups demonstrated significant amplitude differences for positive slow waves [group-by-electrode site: *F* (6, 288) = 6.34, p<0.001, *η*^2^ = 0.112]. Specifically, patients had larger component amplitudes than controls at the Fp2, AF3, and F6 electrode sites (6.52 ± 0.85 *μ*V vs. 1.88 ± 0.78 *μ*V, p < 0.001; 4.73 ± 0.72 *μ*V vs. 2.46 ± 0.66 *μ*V, p = 0.024; 4.77 ± 0.67 *μ*V vs. 2.64 ± 0.62 *μ*V, p = 0.025, respectively.) (Fig. 2(c)). Moreover, positive slow wave amplitudess howed a significant interaction effect of task condition by electrode site [*F* (12, 576) = 4.109, p = 0.001, *η*^2^ = 0.078] (Fig. 3(d)); compared with the other-referential (3.88 ± 0.68 *μ*V) and font-judgment (3.29 ± 0.60 *μ*V) task, self-referencing (5.42 ± 0.65 *μ*V) evoked larger positive slow wave amplitudes (p = 0.001, p = 0.001, respectively at Fp2).

#### Correlation analyses

Recognition scores for the self-referential condition in the patient group correlated significantly with mean positive slow wave amplitudes [*r* (23) = 0.45, p = 0.031, at electrode site AF3; *r* (23) = 0.58, p = 0.008, at electrode site F5]. No significant correlations were found in the control group. Moreover, greater YMRS scores were associated with decreased SRM bias scores [*r* (23) = −0.425, p = 0.043] in the patient group, and there were no correlations between HAMD scores and the behavior measurements.

### Effects of bipolar subtype

To investigate the extent to which psychosis may have an effect on SRM task performance in patients with bipolar disorder, we conducted repeated-measures ANOVAs to compare bipolar I patients with a history of psychosis, bipolar I patients without a history of psychosis, and the control group on both behavioral and ERP results. There were no significant differences across the three groups with respect to age [*F* (2, 49) = 1.05, p = 0.357], sex (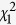 = 2.18, p = 0.336), or years of education [*F* (2, 49) = 1.44, p = 0.247].

**Figure 4:**
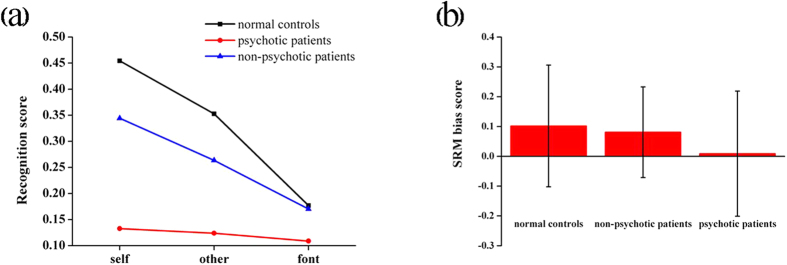
Behavioral results forbipolar disordersubtype and healthy control subjects. (**a**) Recognition scores in the three experimental conditions. (**b**) The self-referential memory (SRM) bias scores. Error bars correspond to two standard deviations of the mean.

The three groups demonstrated significant differences on recognition scores [group-by-task condition: *F* (4, 94) = 8.40, p < 0.001, *η*^2^ = 0.166](Fig. 4(a)). Specifically, the controls performed better than psychotic bipolar patients in the self- (0.45 ± 0.03 vs. 0.13 ± 0.05, p < 0.001) and other-referential conditions (0.35 ± 0.02 vs. 0.12 ± 0.04, p < 0.001), whereas there were no significant differences between the two groups for the font-judgment condition (p = 0.10). Similarly, non-psychotic bipolar patients had higher recognition scores in the self- (0.34 ± 0.04) and other-referential (0.26 ± 0.03) conditions than psychotic bipolar patients (p = 0.003; p = 0.040, respectively); however, the two groups had comparable scores for the font-judgment condition (p = 0.241). Both the healthy controls and non-psychotic bipolar patients had higher recognition scores in the self-referential condition than in the other-referential condition (p < 0.001; p = 0.006, respectively), reflecting a reliable SRM effect, which was not found in psychotic bipolar patients (p = 1.00). In addition, non-psychotic bipolar patients had comparative SRM bias scores (0.08 ± 0.02) to the healthy controls [0.10 ± 0.10, *t* (39) = −0.71, p = 0.482], but psychotic bipolar patients (0.01 ± 0.03) had smaller SRM bias scores than did controls [*t* (34) = −2.464, p = 0.019] (Fig. 4(b)).

**Figure 5:**
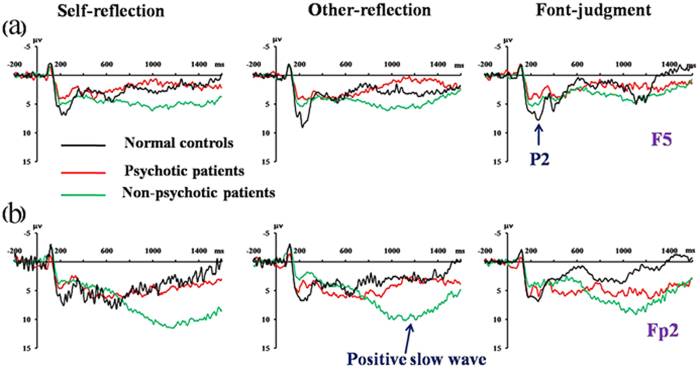
Comparisons of grand averaged waveforms acrosspsychotic, non-psychotic, and healthy control subjects. (**a**) The P2 component at electrode site F5. (**b**) The positive slow waveform at electrode site Fp2.

The three groups demonstrated significant differences in P2 amplitudes [main effect of group: *F* (2, 47) = 5.73, p = 0.006, *η*^2^ = 0.196](Fig. 5(a)), with amplitudes being larger for controls (9.70 ± 0.56 *μ*V) than the two patient groups (psychotic patients: 6.61 ± 0.10 *μ*V, p = 0.024; non-psychotic patients: 7.15 ± 0.78 *μ*V; p = 0.031). The three groups also demonstrated significant differences in mean positive slow wave amplitudes[group-by-electrode sites: *F* (12, 282) = 3.37, p = 0.002, *η*^2^ = 0.118] (Fig. 5(b)), with amplitudes being larger in non-psychotic bipolar patients than in controls across experimental conditions (most prominent at Fp2: 7.74 ± 1.06 *μ*V vs. 1.88 ± 0.77 *μ*V, p < 0.001). However, there were no significant differences between psychotic bipolar patients and controls and nonpsychotic bipolar patients (p > 0.05).

## Discussion

The current study evaluated self-referential processing, an important sub-domain of social cognition, in patients with bipolar disorder. The present results provide evidence that self-referential processing is impaired in patients with bipolar disorderfrom both behavioral and electrophysiological perspectives.

Behaviorally, the two groups demonstrated significant differences in recognition scores, such that patients with bipolar disorder had significantly lower recognition scores in both the self-referential and other-referential conditions. This suggests that both self-referential and other-referential processes are impaired in bipolar disorder. Moreover, the SRM effect for patients with bipolar was associated with YMRS scores; the more serious the manic mood, the SRM bias decreases. Our findings are inconsistent with prior research conducted by Lee *et al*.^17^. Data in their study indicated that patients with bipolar disorder did not differ significantly from comparison subjects during self- and other-referential conditions. This discrepancy may have emerged for several reasons. Firstly, the patient groups in their study were in remission, while patients with bipolar disorder in the present study had higher mania scores. According to our present results, clinical symptoms (YMRS) may have had an effect on the SRM effect, which indicated that disrupted self-referential processing might be state-dependent. Moreover, both healthy controls and patients with bipolar disorder did not display an SRM effect in Lee and colleagues’ study; thus, their results may have been affected by the validity of their experiment paradigm.

From a neurophysiological perspective, we first observed that patients with bipolar disorder had reduced N1 amplitudes compared to healthy controls. The N1 component is an index of early visual processing^13^,^18^, and smaller N1 amplitudes suggest that early visual processing is impaired in bipolar disorder. Additionally, the self-referential N1 was larger than the other-referential N1 in bipolar disorder, while there were no significant differences in N1 amplitudes between the two conditions in controls. This result in the control group was consistent with previous reports in healthy subjects, indicating that early visual processing is similar during the two conditions^18^. The N1 reflects enhanced attention to attended or task-related stimuli^19^,^20^; thus, the findings in patients with bipolar disorder suggested that stimuli in the self-referential condition required more attentional resources for subsequent information processing than in the other-referential condition. Secondly, our results showed reduced frontal P2 activity in patients with bipolar disorder compared to controls. The P2 is related to perceptual analysis, indexes rapid detection of typical stimulus features, and is modulatedby attentional recruitment^21^,^22^. Larger P2 amplitudes in healthy controls might reflect the recruitment of enhanced attentional resources toward self-and other-referential stimuli compared to patients with bipolar disorder, due to the biological importance of these stimuli.

Moreover, larger P3 amplitudes were elicited in the self-referential conditioncompared to the other-referential condition in controls but not in patients with bipolar disorder. Consistent with previous reports^23^,^24^, our results indicate that an SRM effect may have occurred during the P3 component in healthy controls. The P3 component is the most noticeable marker of the self-referential effect^18^, is related to attentional resource allocation^25^, and reflects making yes/no decisions^13^. Larger P3 amplitudes suggest that self-referential stimuli recruit a larger amount of attentional and cognitive resources than other-related stimuli in controls. However, the primacy of self-referential versus other-referential processing did not take place at the P3 processing stage in patients with bipolar disorder.

Fourthly, the positive slow waves during 600–1, 600 ms post-stimulus onset reflect the integration of self-relevant information, a complex interaction of cognitive and emotional processing following basic cognitive processing. Positive slow wave amplitudes at prefrontal electrode sites were larger in patients than in controls. Moreover, according to the subgroup analysis, the current study observed that the mean positive slow wave amplitudes were larger in patients with non-psychotic bipolar disorder than in controls. However, there were no significant differences between patients with psychotic bipolar disorder, non-psychotic bipolar disorder, and controls. Meanwhile, patients with non-psychotic bipolar disorder had a comparatively normal SRM effect, which was abolished in the patients with psychotic bipolar disorder. This suggests that self-referential processing deficits might only emerge in patients with psychotic bipolar. Both of the two patient groups exhibited reduced P2 amplitudes indexing diminished automatic attention capture and orienting toward the self- and other-referential stimuli relative to control subjects^26^. This augmented processing of self- and other-referential information, indexed by larger positive slow wave amplitudes in patients with non-psychotic bipolar disorder, may compensate for the initial insensitive attention toward self- and other-referential stimuli. This might have contributed to the manifestation of an SRM bias. However, in patients with psychotic bipolar disorder, this compensation might have been diminished and resulted in an abolished SRM effect.

As the sample sizes of the two bipolar patient groups were relatively small, these findings should be interpreted with caution. Moreover, as there was no significant difference in symptom severity (5.11 ± 1.05 vs. 5.14 ± 1.41, p = 0.954; 3.93 ± 3.55 vs. 2.50 (16), p = 0.949; 20.96 ± 9.35 vs. 16.37 ± 11.18, p = 0.319, respectively for overall severity, HAMD, and YMRS scores) between the two patient groups, perhaps psychotic features, rather than state-dependent symptom severity, are the main factor contributing to divergent results between the two patient groups. Future work with larger groups of patients with psychotic and non-psychotic bipolar disorder, along with patients with schizophrenia, may allow powerful empirical assessments of whether self-referential processing deficits are discriminating features of major psychotic disorders. This should have important implications for differential diagnoses. Additionally, the positive correlation between the mean positive wave amplitudes and self-referential recognition scores in patients with bipolar disorder provides a direct connection between brain activity during strategic processing of self-relevant emotional information and relevant memory performance^26^.

The present study has a few notable limitations. Firstly, the current sample included hospitalized in-patients; whether the present findings can generalize to euthymic outpatients is unclear and should be addressed in the future. Additionally, although no significant correlations between antipsychotic dosage and behavioral/ERP results emerged, the current patterns should be replicated in non-medicated samples to minimize the confounding effects of medication use.

The present study is an extension of prior research, revealing that self-referential processing deficits are also present in bipolar disorder. Although a self-referential processing advantage was shown in bipolar disorder, the effect occurred later and was less robust than in a control sample. The abnormal self-and other-referential processing observed during early visual and perceptional processing stages was likely related to the complex interaction between cognitive and emotional processing dysfunction over prefrontal cortical regions during later-stage processing.

## Methods

### Subjects

Twenty-three patients with bipolar disorder I disorder (9 psychotic bipolar patients, 14 non-psychotic bipolar patients) and 27 healthy controls were recruited for this study. All patients were hospitalized at Beijing HuiLongGuan Hospital. Inclusion criteria for patients were: (1) bipolar I disorder diagnosis as determined by the Structured Clinical Interview (SCID) from the Diagnostic and Statistical Manual of Mental Disorders-IV^27^, administered by one psychiatrist and confirmed by another senior psychiatrist; (2) no history of significant neurological or medical problems; (3) IQ > 70 (based on medical records); (4) no history of substance abuse or dependence in the past 6 months;and(5) no electroconvulsive therapy in the past 6 months. Clinical assessment was performed using the 21-item Hamilton Depression scale (HAMD)^28^, the Young Mania Rating Scale (YMRS)^29^, and the Clinical Global Impression-Severity scale (CGI-S)^30^. Among the bipolar group, 22 were on antipsychotics (chlorpromazine equivalents: dose = 125–1,000 mg/day, mean = 429 mg)^31^, 5 were on antidepressants, 3 were on lithium, and 15 were taking valproate (dose = 250–1000 mg/day, mean = 650 mg). The control subjects were recruited through advertisements and word of mouth from the surrounding community or were employees of Beijing HuiLongGuan Hospital. The healthy group was free of any DSM-IV Axis I disorder, had no history of substance abuse or dependence in the past 6 months, and had no first-degree relatives with psychotic or mood disorders. The Research Ethics Committee from Beijing HuiLongGuan Hospital approved the study, andthe study was carried out in accordance with the Helsinki Declaration of 1975. Written informed consent was obtained prior to conducting the study. All subjects were compensated for their participation and had normal or corrected to normal vision. There was no significant difference between the two groups with respect to age, education, or sex. Detailed demographic and clinical characteristicsof the study participants can be found in Table 1.

### SRM task

We used the SRM paradigm previously described by our research group^11^. Briefly, the SRM task encompassed two phases: an encoding phase and a recognition phase. A total of 310 personality-trait adjectives (155 positive and 155 negative adjectives) were presented. A total of 210 words were used during the encoding phase, and these 210 words plus the remaining 100 words were used during the recognition phase.

There were three judgment tasks during the encoding phase, and subjects were asked to judge whether a personality-trait adjective was appropriate for describing themselves, the former chairman of the People’s Republic of China (Jintao Hu), or to determine the word font (bold or not). There were 70 words (35 positive and 35 negative words) in each condition during the encoding phase. Each trial started with a 600–1,000 ms fixation cross, followed by a trait adjective with a maximum duration of 4,000 ms. When the subject indicated their response, the next trial was presented 1,000 ms later. After the encoding stage, subjects watched an irrelevant movie for 40 min and were then given an unexpected recognition task. Here, subjects had to answer whether the word was seen during the encoding phase.

### Behavioral measures and analyses

Both stimulus presentation and data recording were conducted using E-Prime 2.0 software (Psychology Software Tools, Inc., USA). Data analyses were performed using SPSS Statistics 16.0 (IBM, USA).

For the encoding phase, mean response time (RT) was calculated for each of the three task conditions, and a two-way ANOVA for subjects’ RTs was conducted with group (patients/controls) as the between-subjects factor and task condition (self/other/font) as the within-subjects factor. For the recognition phase, we mainly calculated two variables: One was recognition score, which was defined as the proportion of hits minus the proportion of false alarms in each condition^4^,^10^,^32^, and the other was an SRM bias score, which was defined as the differential recognition score between the self- and other-referential conditions^2^. A two-way ANOVA with task condition and group as factors was performed to compare group differences on recognition scores, and an independent-samples *t*-test was conducted to compare mean SRM bias magnitudes between the groups. Eta-squared (*η*^2^) was reported to index the effect size in ANOVAs.

### Electroencephalogram (EEG) recording and analyses

Brain electrical activity was recorded from 64 scalp sites using tin electrodes mounted on an elastic cap (Brain Product), with the reference on the left mastoids and a ground electrode on the medial frontal aspect. The vertical electrooculogram (EOG) was recorded with electrodes placed above and below the left eye. The horizontal EOG was recorded as the left versus right orbital rim. The EEG and EOG signals were amplified with a 0.01–100 Hz bandpass filter and were continuously sampled at 500 Hz/channel. Impedance was kept below 5 kΩ. The EEG data were analyzed for ERPs with the Brain Vision Analyzer software (Brain Products). Averaging of ERPs was computed off-line. The EEG data were re-referenced to the algebraic average of the electrodes at the left and right mastoids, and ERPs were filtered with a high-frequency cutoff of 30 Hz (roll-off, 24 dB per octave) before further processing. Epochs were baseline corrected using mean voltage activity during 200 ms prestimulus period. Trails containing behavior errors (i.e. no response was made/incorrect font-judgment) and blinks, eye movements, or other artifacts (EEG sweeps with amplitudes exceeding ± 100 *μ*V) were excluded from averaging. The mean (SD) % of trials rejected for each condition in controls was: self-reflection = 3.58% (4.59), other-reflection = 3.23% (4.00) and font-judgment = 4.70% (4.91) and that in bipolar patients was: self-reflection = 4.63% (5.32), other-reflection = 5.55% (7.15) and font-judgment = 7.95% (7.70). Chi-square Tests indicated that there were no significant differences in terms of rejected trials between patients and controls (p = 0.55, p = 0.913, p = 0.180, respectively for self-reflection, other-reflection, and font-judgment condition).

EEG activity during the encoding stage was overlapped and averaged separately in each condition and for both groups. ERP waveforms were time-locked to stimulus onset, and the average epoch was 1,800 ms, including a 200 ms pre-stimulus baseline. According to the ERP’s grand averaged waveforms and topographical map, peak latencies (from stimulus onset to the peak of each component) and amplitudes (N1, P2 and N2: baseline to peak; P300 and the positive slow wave: the average amplitude) of five components showed different latencies and/or amplitudes across task conditions, and/or groups were measured. The ERP components were analyzed across different sets of electrodes according to both the ERP topographies and relevant literature^17^,^26^,^33^,^34^. In particular, the P7, P8, PO7, and PO8 were selected for statistical analysis of the N1 component (150–220 ms); P2 (130–320 ms) and the positive slow waves (600–1,600 ms) were calculated at Fp1, Fp2, AF3, AF4, F5, Fz and F6 electrode sites; the N2 component was measured at F3, Fz and F4 electrode sites (270–400 ms); and the P3 component (320–600 ms) was calculated at P1, P2, Pz, CP3, CPz and CP4 electrode sites.

A three-way ANOVA was conducted to assess differencesin all dependent measurements (N1, P2, N2, P300, and the positive slow wave amplitudes and latencies) between groups, across conditions, and at different electrode sites. The Greenhouse-Geisser correction was used to compensate for sphericity violations. Pearson’s correlation coefficients were calculated to assess relationships between ERP measurements and behavioral performance during the recognition phase, as well as the associations between behavioral performance and mood (YMRS/HAMD) in the patient group. Correction for multiple comparisons was based on Holm’s stepwise correction. A two-tailed p value of <0.05 was used for significance testing.

## Additional Information

**How to cite this article**: Zhao, Y. *et al*. Behavioral and neural correlates of self-referential processing deficits in bipolar disorder. *Sci. Rep*. **6**, 24075; doi: 10.1038/srep24075 (2016).

## Figures and Tables

**Table 1:** Demographic and clinical data for patient and control groups.

	Group; mean ± SD or no. (%)*		Comparison; P value
characteristic	Patients (n = 23)	Controls (n = 27)	Patients v.controls
Age (years)	34.3 ± 12.0	32.3 ± 8.64	0.497
Education time (years)	12.6 ± 3.22	13.9 ± 2.50	0.110
Gender (female)	11(48)	14(52)	0.777
Handeness (right)	23(100)	27(100)	
Age at onset (years)	23.8 ± 8.30		
Days of hospital stay	14(17)†		
Duration of illness (years)	10.5 ± 9.05		
Current symptomatoloty			
CGI-S	5.13 ± 1.25		
Depression (HAMD)	6.2 ± 8.61		
Mania (YMRS)	18.2 ± 10.53		
Medication			
valproate	15(65)		
antidepressants	5(22)		
antipsychotics	22(96)		
lithium	3(13)		
	Psychotic bipolar patients (n = 9)	Non-psychotic bipolar patients (n = 14)	Psychotic patients vs. non-psychotic patients
Age (years)	37.7 ± 11.0	32.1 ± 12.5	0.285
Gender (male/female)	3/6	9/5	0.214
Education time (years)	12.2 ± 2.9	12.9 ± 3.5	0.655
Days of hospital stay	19.6 ± 12.0	20.9 ± 22.1	0.866
Illness duration (years)	9.44 ± 4.16	4.50(16.75)†	0.776
CGI-S	5.11 ± 1.05	5.14 ± 1.41	0.954
HAMD	3.93 ± 3.55	2.50(16)†	0.949
YMRS	20.96 ± 9.35	16.37 ± 11.18	0.319
Medication dosage			
valproate	500(500)†	500(500)†	0.572
antipsychotics	250(175)	423.89 ± 293.65	0.227

^*^Unless indicated otherwise.

^†^Median (interquartile range) and significance was determined with the nonparametric Mann–Whitney *U* test.
